# Novel Results and Concepts Emerging From Lipid Cell Biology Relevant to Degenerative Brain Aging and Disease

**DOI:** 10.3389/fneur.2019.01053

**Published:** 2019-10-09

**Authors:** Ole Isacson, Oeystein R. Brekk, Penelope J. Hallett

**Affiliations:** McLean Hospital and Harvard Medical School, Neuroregeneration Research Institute, Belmont, MA, United States

**Keywords:** neurons, lipids, astrocytes, microglia, inflammation, α-synuclein, apolipoprotein, Parkinson's disease

## Abstract

While very rare familial forms of proteinopathy can cause Parkinson's disease (PD), Lewy body dementia (LBD) and age-related dementias, recent in-depth studies of lipid disturbances in the majority of the common forms of these diseases instead suggest a primary pathogenesis in lipid pathways. This review synthesizes a perspective from new data that point to an interdependence of lipids and proteinopathy. This article describes disturbed relationships in lipid homeostasis that causes neuropathology to develop over time and with age, which includes altered mechanisms of glia-neuron exchange of lipids and inflammatory signals.

## Lipid Changes in Parkinson's Disease and Related Neurodegeneration

The relationship between lysosomal storage disorders (LSDs) and Lewy body disorders became apparent through evidence of increased risk for developing Lewy body dementia (LBD) and Parkinson's disease (PD) in carriers of LSD gene mutations, and through glycosphingolipid dysregulation and lysosomal dysfunction implicated in the normal pathophysiology of PD (1, 2) (Table 1). Homozygous mutations in the lysosomal hydrolase glucocerebrosidase, *GBA1*, are associated with the LSD, Gaucher disease. Haploinsufficiency of *GBA1*, which causes reduced activity of glucocerebrosidase (GCase), is associated clinically with a significantly increased risk of PD and LBD, and with faster rate of cognitive decline in α-synucleinopathies, including LBD and PD (3, 4). Brain GCase activity is also decreased and corresponding glycosphingolipid substrate levels are elevated in the brain in PD without *GBA1* mutations (1, 5–7) pointing to a much broader age-related decline, and to more complex mechanisms (8). Similar dysfunction of lysosomal hydrolases and disturbances in glycosphingolipid levels to those found clinically in PD are observed in normal aging of both mouse and human brain (1, 9).

**Table 1:** Biochemical and clinical evidence for lysosomal enzyme loss of function and lipid disturbances creating Parkinson's disease-like pathology.

**Relevant lysosomal biochemical pre- and clinical data for parkinson's disease and related disorders**	**Protein affected**	**Accumulating substrate(s)**
Increased risk for PD and LBD in patients carrying *het*erozygousĞBA1 mutations. Glucocerebrosidase activity is reduced in sporadic PD and in normal aging. GluCer and GluSph increased in sporadic PD brain (1).	Glucocerebrosidase	Glucosylceramide and glucosylsphingosine
LIMP-2, which transports GCase to lysosome, is encoded by SCARB2. SCARB2 gene variants are associated with PD and LBD risk (11, 12). LIMP-2 deficiency in mice causes GCase activity reduction, glycolipid accumulation and αSyn aggregates (13).	Glucocerebrosidase	Glucosylceramide
Granulin (GRN) gene variants associated with PD risk (14). Progranulin deficiency in mice leads to reduced GCase activity (15).	Glucocerebrosidase	–
Clinical reports of parkinsonism in Fabry disease patients (16). Reduced activity of α-galactosidase A in leukocytes of PD patients (17).	α-galactosidase A	Globotriasylceramide
Generalized dystonia associated with akinetic-rigid Parkinsonism reported in patients with GM1 gangliosidosis (18) (caused by a β-galactosidase deficiency). Lactosylceramide is utilized in ganglioside biosynthesis.	β-galactosidase	Lactosylceramide
Mutations in SMPD1 lead to Niemann-Pick disease type A or B and accumulation of sphingomyelin, and are also associated with increased risk for PD (19).	Acid sphingomyelinase	Sphingomyelin
Phosphorylated αSyn and Tau in neurons and oligodendrocytes in Niemann-Pick disease type C patient brain (20).	NPC1 and 2	Cholesterol, sphingolipids
α-synucleinopathy reported in the brain of patients with Krabbe's disease (21).	Galactocerebrosidase	Galactosylsphingosine
α-synucleinopathy reported in brain of patients with Sandhoff disease, as well as in Hexb deficient mice (22, 23). Parkinsonism reported in patients with Sandhoff disease (24).	β-hexosaminidase A and B	GM2 ganglioside
Variation in NAGLU associated with risk for PD. Intracellular αSyn accumulation in cortical tissue from Sanfilippo A syndrome cases (25).	N-acetylglucosaminidase, N-sulfoglucosamine sulfohydrolase	Heparin sulfate metabolites

Furthermore, PD gene expression and genetic analyses of large cohorts also point to an early involvement of biological processes *upstream* of accumulating alpha-synuclein (αSyn), including involvement of lipids and lipoproteins, oxidative stress, endosomal-lysosomal functioning, endoplasmic reticulum stress, and immune response activation (8, 10). In addition, with age, lysosomal enzyme function, chaperones and transporters present in the endoplasmic reticulum-Golgi complex, may become compromised at an early stage of pathogenesis. Critically, biochemical evidence shows that lysosomal enzyme loss of function and lipid disturbances creates PD-like pathology (see Table 1). With age, many mechanisms can compromise lysosomal enzyme function, including loss of chaperones and transporters present in the endoplasmic reticulum-Golgi complex. Insufficiency of such lysosomal enzymes puts certain cells and brain regions at risk over the longer time frame associated with a relative increase of longevity in humans, creating risk for age-related neurodegenerative diseases (8).

Over the last decades there has been a large emphasis placed on the idea of “proteinopathy”, conceptualized primarily in two versions. First, as a primary mechanism for cell dysfunction and degeneration in PD and other diseases with cellular protein aggregates, which however may only be true in very rare genetic cases with gene copy number variations (CNVs) or rare mutations (26). For example, whilst familial PD cases with CNVs of αSyn (duplication, triplication) cause genetic PD and LBD and protein elevations, such cases are very rare; there are currently ~80 individuals worldwide carrying these CNVs diagnosed with PD/LBD (26), compared to an estimated 6 million cases of PD worldwide (27). Second, the theory of causative proteinopathy has been extended to encompass extra-neural spread of toxic proteins in order to explain regional patterns of chronic cellular pathology seen in many neurodegenerative disorders. Evidence of physical αSyn spread between cells in human PD and related diseases remains to be established, and so far is demonstrated only in artificial experimental model systems.

Instead, more obvious causes for cell dysfunction and pathologies in PD and other related disorders, are *primary* disturbances from lipids and other metabolic stressors, which in turn can produce protein elevation and aggregation. Lewy body inclusions, widely believed to be predominantly composed of proteinaceous filaments, are in fact more co-labeled with lipids (28, 29). Importantly, recent ultrastructural findings demonstrate that Lewy bodies and neurites in PD post-mortem brain are composed of abundant membranous structures, abnormal vesicles and autophagosome-like structures, in addition to disrupted cytoskeletal elements and dysmorphic mitochondria (30). In summary, the previous almost exclusive focus on aggregating proteins in familial and sporadic cases of PD and LBD, may be replaced by a critical analysis of intracellular lipids and dysfunctional lipid transport as primary mechanisms of disease; in concert with inflammatory processes for PD and LBD. Such analyses may be very useful in the future for selecting candidates, biomarkers and modalities for treatments.

How can perturbation of glycolipid metabolism and lysosomal homeostasis in the aging brain precede or be upstream of protein handling? Glycolipids are abundant in plasma and intracellular membranes and are particularly enriched in the brain where they act as cell surface recognition molecules, as well as having essential roles in the regulation of membrane fluidity and lipid raft formation, modulation of membrane-protein function and signal transduction (31). When GCase in inhibited *in vivo*, there is large accumulation of high molecular weight aggregates of αSyn (32) and *in vivo* genetic models of primary GBA mutations also produce significant α-synucleinopathy over time (33, 34). These α-synucleinopathies can be reversed or prevented by agents that increase GCase, or otherwise reduce the accumulation of glycolipids (33, 35). In conclusion, the physiological burden of elevated neuronal glycolipid load in aging and in PD/LBD would affect multiple organelles and biological pathways, and may lower the threshold for developing PD and related neurodegenerative disorders, or accelerate pathophysiological processes in vulnerable neurons.

## Neuron-Glia Interactions During Lipid Dyshomeostasis

**Figure 1:**
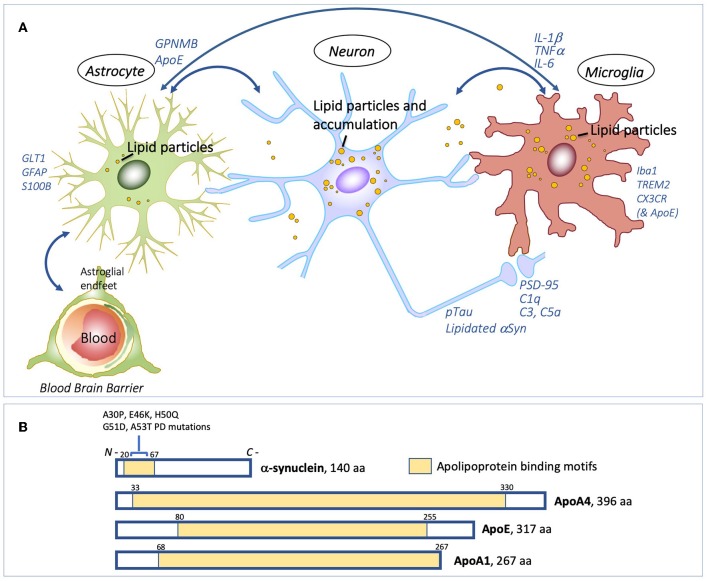
Interdependence of lipid processing and proteinopathy. Lipids, and glia-neuron exchange of lipids and inflammatory signals **(A)** Proposed model for pathogenic lipid perturbations in human PD. Overall accumulation of glycolipids in the substantia nigra with differentially altered lipid droplet deposition in neurons and glial cells could facilitate aberrant protein-lipid interactions (e.g. with αSyn and pTau), in turn perturbing the neuron-glia lipid exchange and activating GPNMB inflammatory protein signals. Cellular dysfunction caused by elevated glycolipids could converge on downstream cytokine-signaling, and other immune responses, in neurons, microglia and astrocytes, causing excessive and aberrant neurite and synaptic damage ultimately leading to neurodegeneration. **(B)** αSyn has a role in lipid binding through its 11-amino acid repeat amphipathic helical region (shaded in yellow). Mutations in αSyn associated with familial PD, A30P, E46K, H50Q, G51D, and A53T, all occur within this region. αSyn shares conserved tandem repeat regions with the apolipoproteins, ApoA4, ApoE, and ApoA1 (yellow shading). Under lipid stress or aging, αSyn can become significantly lipidated, which may also create dysfunction leading to Lewy bodies. Schematic in **(A)** is original by the authors. Schematic in **(B)**, showing apolipoprotein binding motifs, is adapted and modified from Emamzadeh (38), under terms of the Creative Commons CC BY license.

Abnormal glycolipid, neutral lipid and protein homeostasis within PD and LBD susceptible neurons are likely to signal to surrounding cells, including microglia and astrocytes, accelerating neurodegeneration (36, 37) (Figure 1A). Understanding how glycolipid changes can drive the inflammatory and neurodegenerative mechanisms will be crucial in enabling the development of novel therapeutics. Elevation of reactive oxygen species, mitochondrial dysfunction and loss of autophagy in neurons leads to elevated lipid particle formation (and peroxidated fatty acids). Such lipid dyshomeostasis in neurons may lead to subsequent accumulation of lipid droplets and eventually larger undigested lipid particles, in neurons, and potentially neighboring microglia (39–41) and Isacson, Brekk, Hallett *unpublished observation*. Appropriate lipid transfer between these cells via lipid transporters such as apolipoproteins, is essential for this process, and for maintaining metabolic integrity of the neuron (40). Peroxidated fatty acids released by neurons are bound to lipoproteins, which are endocytosed by glia (41). Disrupting the transport of lipids from neurons to glia for lipid droplet formation under conditions of neuronal stress, leads to neurodegeneration (39).

Glycoprotein non-metastatic protein B (*GPNMB*) is a type-I transmembrane glycoprotein that seems to be mechanistically related to altered glycolipid levels (42). In the brain, GPNMB is expressed primarily in glial cells and is thought to play a role as an inflammatory regulator to prevent chronic inflammation (43). Polymorphisms in *GPNMB* are found to associate with idiopathic PD (44), and GPNMB protein levels are selectively increased in the substantia nigra of PD patients (42). In Gaucher disease, increased protein levels of GPNMB correlate with disease severity and progression (45, 46). Upon systemic pharmacological induction of lipidopathy in mice, which also causes α-synucleinopathy, GPNMB is elevated in a brain-region specific manner including the hippocampus, substantia nigra and cerebral cortex (42). This regional specific upregulation of GPNMB may reflect a differential response of brain regions to lysosomal dysfunction and subsequent differential vulnerability of neuronal populations to degeneration (42, 47). In this lipidopathy model, GPNMB elevation is also associated with robust glial activation and GPNMB is localized in astrocytes and microglia (42). Of note, GPNMB is not elevated by increased αSyn alone in transgenic mice overexpressing human αSyn, indicating a selective response to lipid perturbations (42).

## αSyn as a Lipid-Carrying Protein

αSyn is abundant in neurons and is highly enriched at presynaptic terminals, and it is also associated with some organelles. αSyn has important functional roles in the regulation of vesicles, such as synaptic vesicles, neurotransmitter release, dopamine metabolism, synaptic activity, and plasticity (48). αSyn can interact with various lipid species through its amphipathic N-terminus (amino acid) domain. The N-terminal region of αSyn contains six hexameric repeats of 11 amino acids which are characteristic of the 11 amino acid repeats that mediate lipid interactions of apolipoproteins (38, 49) (Figure 1B). Significantly, all mutations in αSyn which are associated with familial PD, are located in this lipid-binding region. In the brain, apolipoprotein E (apoE) is the most abundant lipoprotein, forming lipoprotein particles and binding to ApoE cell surface receptors for the delivery of cholesterol and other lipids to neurons. The *APOE4* isoform allele is associated with increased risk for dementias in Lewy body diseases, including LBD and PD (50). Interestingly, plasma protein levels of another apolipoprotein, apolipoprotein A1, are associated with age of onset and motor severity in early PD (51). αSyn has several apolipoprotein-like characteristics, including regulation of cholesterol efflux in neuronal cells and formation of lipoprotein nanoparticles (52, 53) providing a clear premise for αSyn's function as a lipid-carrying molecule. Statin treatment in a rodent α-synucleinopathy model reduces α-synuclein aggregates and neuronal pathology (54). The role of αSyn as a functional apolipoprotein is also highlighted by the finding that there is a worsening of ApoE4-mediated pathology in mice that carry human apoE4 when mouse αSyn is ablated (55). Levels of neutral lipids are elevated in the brain in mice that lack endogenous αSyn (56). In perspective, given these data it appears that αSyn normally is not participating in lipid transport in roles that involve synaptic transmission and vesicular functions at the synapse, but can become lipidated under cellular stressful conditions that involve glycolipid, sphingolipid, neutral lipid, lipid peroxidation and age-related disturbances (7). In such circumstances, αSyn becomes part of a pathological adaptation to resolve the lipid problems (55), which likely leads to vesicular binding and transport changes that precede Lewy body formation.

Apolipoprotein function is also linked with inflammation, and ApoE is a modulator of immune responses (57) (Figure 1A). Mice lacking ApoE show similar immune activation to mice expressing human ApoE4 in response to lipopolysaccharide (58), and expression of complement pathway genes are upregulated in ApoE knockout mice (59). ApoE has also recently been shown to form a complex with C1q within lipid compartments where it is a regulator of the classical complement cascade (59). C1q is implicated as an early mediator of neuronal dysfunction in preclinical models of AD, whereby reduced expression or blockade of C1q rescues synaptic loss and dysfunction upon exposure to toxic amyloid-beta (60); similarly, C1qa knockdown mitigates neurotoxicity in an *in vivo* model of frontotemporal dementia (61). Furthermore, C1qa deficiency delays functional cognitive decline associated with normal aging in mice (62). Activation of the complement system is induced in lysosomal storage disorders, including models of neuronopathic Gaucher disease by inhibiting GCase (using CBE), where protein expression of C1q is robustly elevated in several brain regions (32). Inhibition of the complement pathway, through genetic deficiency of C5R1a completely prevents glycolipid accumulation and inflammation in the brain following similar paradigms of systemic CBE (63).

## Summary and Future Perspective

**Figure 2:**
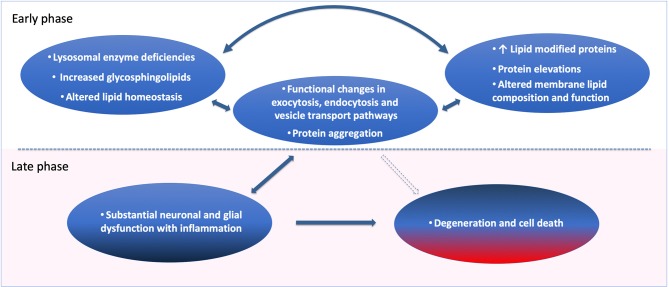
Cell biological pathogenic factors and adaptations leading to neuronal dysfunction, synaptic degeneration, and cell death over time. Loss of lipid homeostasis and lysosomal enzyme deficiencies are the strong genetic and phenotypic drivers of age and Parkinson's disease. Sphingolipid elevations and changes in lipid metabolism can be (Early, see figure) primary pathogenic contributors, leading to protein elevations, lipid modified proteins and sphingolipid-induced membrane composition and loss of function. In the case of CNVs and in mutations of key proteins such as αSyn, the primary event in those extremely rare forms of PD can start with protein elevations followed by altered lipid-modified proteins, and functional changes in exocytosis, endocytosis and vesicle transport, leading to neuronal dysfunction and protein aggregation. It is reasonable to believe that these cellular changes can occur for a long time in an adaptive feedback cycle to keep the cells and neurons functional, as a form of pathological accommodation. Over time with the ongoing disease process (Late, see figure), the cell dysfunction will become severe if no external intervention is obtained, causing substantial neuronal glial dysfunction and inflammation as a result. Such conditions will lead to regional and cell type specific destruction and cell death in the brain tissue.

In summary, much evidence points to disruption of lipid cell biology; as glycosphingolipids, gangliosides and possibly several other lipids with metabolic influence can be early initiating factors for age-related neurodegenerative disorders such as PD and LBD (Table 1 and Figure 2). Lipid disturbances in cell types of the brain and/or in specific compartments of such cells, including neurons, astrocytes, microglia and oligodendrocytes, are involved in a large number of neurological diseases. In particular, it is now clear that PD can be triggered by lipid disturbances that are caused by lysosomal genetic or similar age-induced enzymatic loss of function. Relevant to such lipid changes, we find that lipid transport may be compromised by pathological accommodation of αSyn to lipid binding and altered transport roles which are not optimal for normal neuronal function (Figure 1). In particular, it is important in future research to identify lipid binding and abnormal lipid droplet or other cellular lipid formations under specific cell biological conditions. Lipid storage diseases with excessive lipid handling demand can lead to astrocytic and microglial disturbances. There are several contexts in which such lipid and associated lipid-protein interactions could eventually become pathological. The fact that the lipid-carrying *APOE4* variant is associated with increased risk for AD and dementias may be the most explicit biological situation where apolipoprotein functions are a major driver of brain dementias. Under some conditions, αSyn may even have a cooperative role with apolipoproteins and lipid transport. In addition, basic research demonstrates that several proteins including αSyn (Figure 1B) can accommodate pathological lipid disturbances in astrocytic, neuronal and microglial compartments. In such situations, for a time affected cells will handle genetic and age-acquired lipid and metabolic disturbances, and clearly, such cells may even return to a healthier condition when the pathological stimuli are removed, or a treatment is devised that addresses the cause or initiating factor. Regardless, in chronic neurodegenerative diseases when neurons and glia are unable to maintain such cellular component functions, the pathogenic mechanisms will lead to cellular functional failures that are irreversible (see Figure 2). Inflammatory responses can be present at any of these pathogenic steps but are potentially most damaging in the later stages of degeneration, as such processes can remove cellular structures, including synapses, permanently at a structural level (Figure 2). The continuous expression of elevated amounts or aberrant lipids inside and outside neurons and glia can activate the immune system. In our opinion, novel research needs to focus on the interactions between neurons and glia as an interdependent system that attempts to regulate lipid and protein changes. When such lipid disturbances are significant they can lead to inflammatory reactions and eventually synaptic pathobiology (Figure 1A).

Lipid dyshomeostasis, transport and clearance are emerging as central causative factors in neurodegenerative diseases and should help in selecting molecular targets for medical treatments, as well as diagnostic insights to both corrective and anti-inflammatory action to prevent structural degeneration in the brain. This new perspective of pathogenesis relevant, upstream causative mechanisms in several neurodegenerative diseases in PD, LBD and potentially also age-related dementias, provides optimism in developing new therapies for these devastating diseases.

The implication for the new understanding presented here; that lipid and inflammatory mechanisms can precede proteinopathies (Figure 2), provides clinical opportunities for identification of relevant and specific lipid and inflammatory biomarkers. It is already possible to measure specific abnormalities from GCase in the blood and brain (1, 64) of patients with PD. Such patient stratification in other lipid species and specific metabolic disturbances may help to better define effective treatments of neurodegenerative diseases in clinical trials. It is also important that systemic and peripheral biomarkers can be coincident with brain pathology observed in neurodegenerative diseases. There is evidence of such peripheral biomarkers in patients with LSDs, PD, and AD for pathways involved in lipids and inflammation (51, 64–68). In conclusion, determining altered cellular lipid accumulation, transfer and clearance mechanisms in PD and related disorders can be of significant value to helping patients and at-risk individuals.

## Author Contributions

OI drafted the article with PH. OB contributed to the discussion and analysis of the results mentioned in the article.

### Conflict of Interest

The authors declare that the research was conducted in the absence of any commercial or financial relationships that could be construed as a potential conflict of interest.
